# Appointment in the University of Pennsylvania

**Published:** 1852-09

**Authors:** 


### APPOINTMENT IN THE UNIVERSITY OF PENNSYLVANIA.

DR. ROBERT E. ROGERS, late Professor of Chemistry in the University of Virginia, has been elected to the same department in the University of Pennsylvania. Dr. Rogers is a brother of the late incumbent, and brings to the chair all the talent and experience that will enable him to fill the post acceptably and profitably.

